# Survival impact and safety of intrathoracic and abdominopelvic cytoreductive surgery in advanced ovarian cancer: a systematic review and meta-analysis

**DOI:** 10.3389/fonc.2024.1335883

**Published:** 2024-01-18

**Authors:** Jiaxi Wang, Xingyu Wang, Wanjun Yin, Shiqian Zhang

**Affiliations:** ^1^ Department of Obstetrics and Gynecology, Qilu Hospital of Shandong University, Jinan, China; ^2^ Weifang Medical University, Weifang, China

**Keywords:** ovarian cancer, intrathoracic cytoreductive surgery, abdominopelvic cytoreductive surgery, residual disease, prognosis

## Abstract

**Purpose:**

Achieving no residual disease is essential for increasing overall survival (OS) and progression-free survival (PFS) in ovarian cancer patients. However, the survival benefit of achieving no residual disease during both intrathoracic and abdominopelvic cytoreductive surgery is still unclear. This meta-analysis aimed to assess the survival benefit and safety of intrathoracic and abdominopelvic cytoreductive surgery in advanced ovarian cancer patients.

**Methods:**

We systematically searched for studies in online databases, including PubMed, Embase, and Web of Science. We used Q statistics and I-squared statistics to evaluate heterogeneity, sensitivity analysis to test the origin of heterogeneity, and Egger’s and Begg’s tests to evaluate publication bias.

**Results:**

We included 4 retrospective cohort studies, including 490 patients, for analysis; these studies were assessed as high-quality studies. The combined hazard ratio (HR) with 95% confidence interval (CI) for OS was 1.92 (95% CI 1.38-2.68), while the combined HR for PFS was 1.91 (95% CI 1.47-2.49). Only 19 patients in the four studies reported major complications, and 4 of these complications were surgery related.

**Conclusion:**

The maximal extent of cytoreduction in the intrathoracic and abdominopelvic tract improves survival outcomes, including OS and PFS, in advanced ovarian cancer patients with acceptable complications.

**Systematic Review Registration:**

PROSPERO, identifier CRD42023468096

## 1 Introduction

Ovarian cancer is the most common gynecological cancer and has a high recurrence rate and mortality rate, with an estimated 21410 new cases and 13770 deaths in 2021 in the United States (1). Currently, a growing number of gynecologic oncologists are investigating the risk factors for survival outcomes in patients with ovarian cancer and are making great efforts to improve patient prognosis. Achieving no residual disease is essential for prolonging overall survival (OS) and progression-free survival (PFS).

Griffiths first demonstrated poor survival time in ovarian cancer patients with more than 1.5 cm residual disease, regardless of the total tumor volume (2). With further research, a number of studies, including randomized controlled trials (RCTs) and meta-analysis, have suggested that resection of all visible tumors is significantly beneficial for survival in patients with ovarian cancer (3–6). With the improvements in surgical practice among oncologists and the support of multidisciplinary care teams, the scope of cytoreductive surgery has changed from abdominopelvic cavity resection to extensive upper abdominal resection, including diaphragm resection, liver resection, splenectomy, pancreatectomy, partial gastrectomy and cholecystectomy, all of which have improved survival outcomes and led to acceptable complications (7, 8). Along with diaphragm resection and video-assisted thoracic surgery (VATS), intrathoracic surgery is no longer a contraindication and is performed as part of primary debulking surgery (PDS) by gynecologic oncologists or thoracic surgeons.

In 2000, Montero et al. reported the first case in which the cardiophrenic lymph node (CPLN) was resected via VATS for ovarian cancer (9). In addition, Pfannschmidt et al. reported that some colorectal cancer patients may benefit from pulmonary metastasectomy (10). Since then, the adoption of intrathoracic surgery for ovarian cancer has increased in gynecologic cancer centers. However, data on the safety and feasibility of thoracic surgery are still limited, and the survival benefit of resecting microscopic intrathoracic disease is still unclear. Therefore, we aimed to conduct a meta-analysis to assess the survival benefit and safety of intrathoracic and abdominopelvic cytoreductive surgery in advanced ovarian cancer patients.

## 2 Methods

### 2.1 Search strategy

This review was registered on PROSPERO (CRD42023468096) and followed the Preferred Reporting Items for Systematic reviews and Meta-Analysis (PRISMA) statement. After searching online databases, including Pubmed, Embase, and Web of Science, until September 2023, we identified the following search terms: “ovarian neoplasm”, “cytoreduction surgical procedure”, “ultraradical”, “thorax”, “mediastinum”, “pleura”, “cardiophrenic lymph node”, “precordial lymph node”, “paracardial lymph node”, “supradiaphragmatic”, “video -assisted thoracoscopic surgery” and “thoracoscopy”. There were no other restrictions in the search strategy. The detailed strategy is shown in **Supplementary File 1**.

### 2.2 Inclusion and exclusion criteria

Studies were included if they met the following criteria: a) had a confirmed pathological examination; b) had advanced ovarian cancer according to the International Federation of Gynecology and Obstetrics (FIGO) stage III-IV disease, underwent both intrathoracic surgery and abdominopelvic cytoreduction surgery and had a record of residual disease; and c) had records on survival outcomes, including OS and PFS, provided with hazard ratios (HRs) and 95% confidence intervals (CIs).

Studies were excluded if they met the following criteria: a) had a comment, letter, case report, conference abstract, review, or meta-analysis; b) lacked data on residual disease and survival outcome; c) had overlapping patients or duplicated published literature; or d) were not written in English.

### 2.3 Data extraction and quality assessment

After using the search terms shown in **Supplementary File 1**, two independent researchers (Jiaxi Wang and Xingyu Wang) extracted each study from the EndNote. If any disagreements occurred, two researchers discussed the study with the third researcher and arrived at a consensus. For each included study, the following data were extracted: author, publication year, country where the study was carried out, study period, sample size, stage, overall survival (OS) and progression-free survival (PFS) with hazard ratio (HR) and 95% confidence interval (CI) and complications.

Two independent researchers (Jiaxi Wang and Xingyu Wang) performed the quality assessment of the included studies according to the Newcastle–Ottawa Scale (NOS), which consists of selection, comparability, and outcome assessment. High-quality studies were considered to have scores greater than 6. Any disagreements were discussed with a third researcher (Shiqian Zhang).

### 2.4 Statistical analysis

The primary endpoint was OS, which was defined as the time from the date of surgery until death or the last follow-up. The secondary endpoint was PFS, which was defined as the time from the date of surgery until the first disease progression, death, or last follow-up. Q statistics and I-squared statistics were used to evaluate heterogeneity. A P>0.05 or I^2^<50% suggested low heterogeneity, and the fixed-effects model was adopted. Otherwise, the random effects model was used. Sensitivity analysis was applied to test the origin of heterogeneity. To evaluate publication bias, we constructed a funnel plot and performed Egger’s and Begg’s tests. P<0.05 was considered to indicate statistical significance.

## 3 Results

### 3.1 Search results

**Figure 1:**
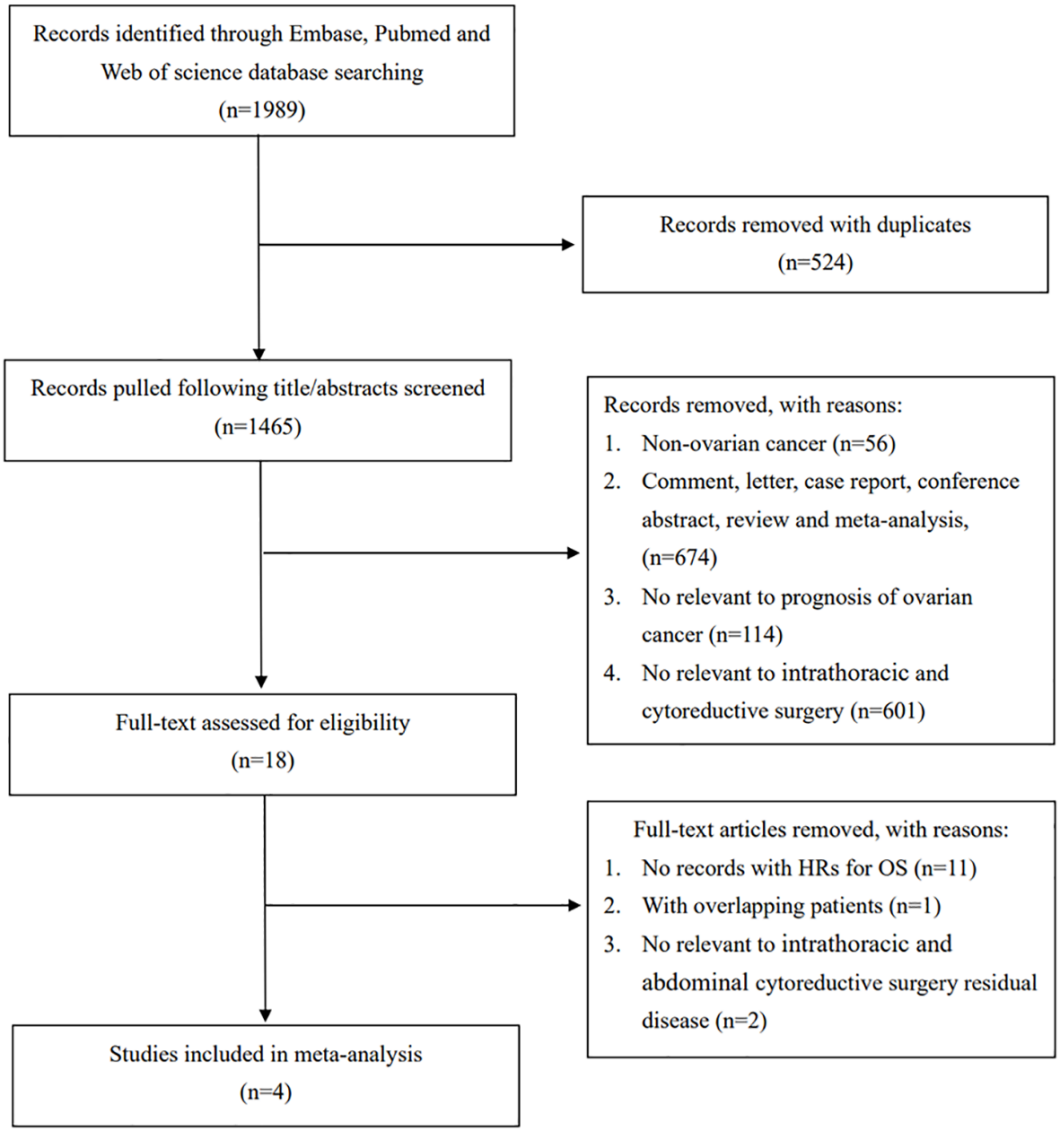
PRISMA flow diagram of the process.

A total of 1989 relevant studies were identified from the database, as shown in **Figure 1**. After excluding duplicate studies, 1447 studies were excluded after screening the title and abstract. The full texts of the remaining 18 studies were assessed, and 4 studies were ultimately included in the meta-analysis (11–14).

### 3.2 Study characteristics and clinical outcomes

**Figure 2:**
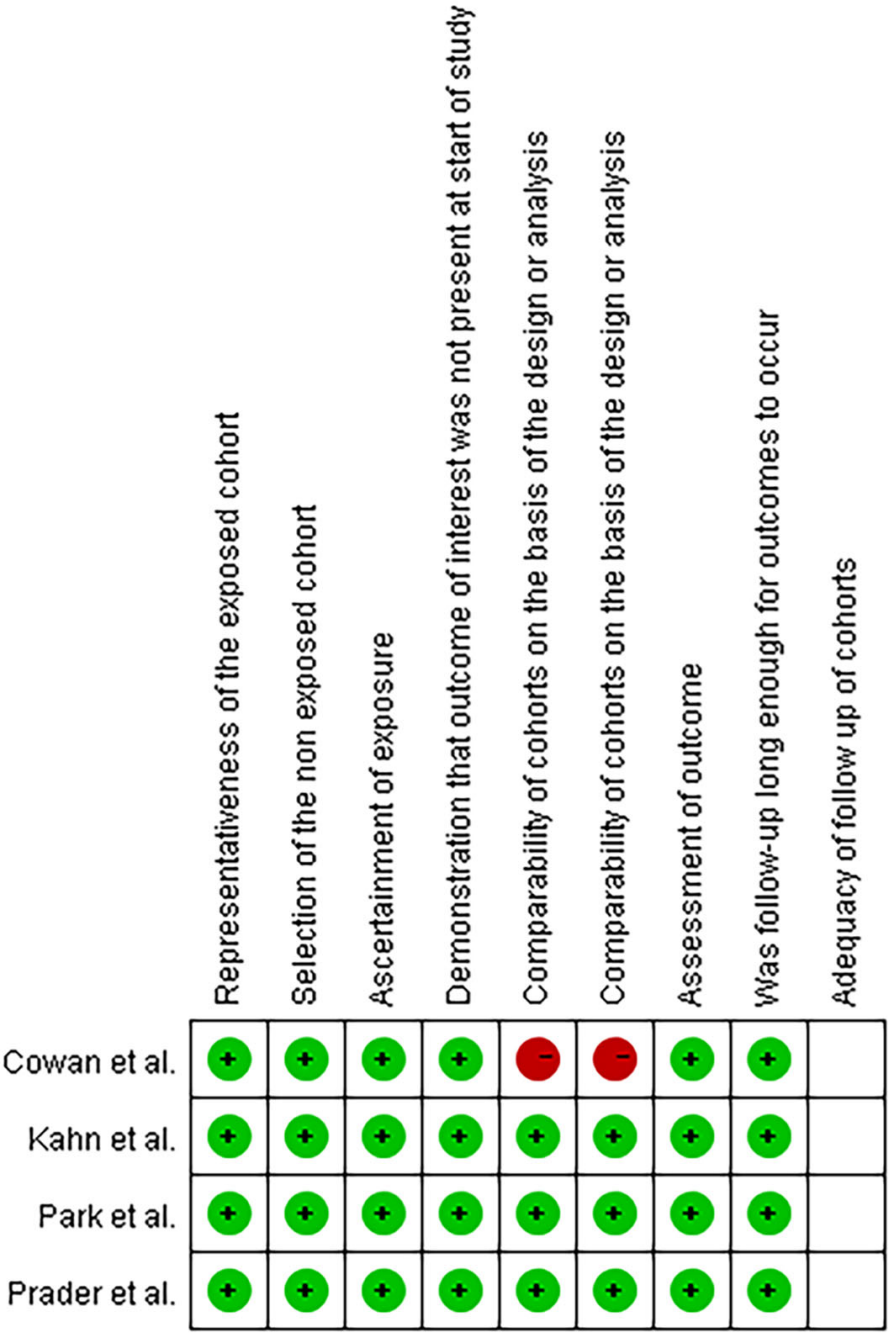
Quality assessment according to the Newcastle-Ottawa Scale (NOS) score.

The four included studies shown in **Table 1** were all retrospective cohort studies carried out in America, Germany, and Korea. The meta-analysis involved 490 advanced ovarian cancer patients who received PDS. The approaches used for intrathoracic surgery included VATS, transdiaphragmatic resection, and the subxiphoid approach. All studies were deemed to be high-quality studies according to the NOS score (**Figure 2**). Major complications were reported in 19 patients among the four studies, and only 4 of these complications were surgery related.

**Table 1:** Main characteristics of the included studies.

Study	Country	Type	Sample	Age	Stage	Treatment	Intrathoracic approach	Outcome	Compli-cations	NOS score
Cowan et al. (2017)	America	retrospective	54	54	IIIB-IV	PDS	A+B+C	OS	19 major complications	6
Prader et al. (2018)	Germany	retrospective	190	NA	IIIB-IV	PDS	B	OS+PFS	No major complications	8
Kahn et al. (2023)	America	retrospective	178	59	III-IV	PDS	A	OS+PFS	No major complications	8
Park et al. (2023)	Korea	retrospective	68	34	IVB	PDS	A+B	OS+PFS	No major complications	8

PDS primary debulking surgery, A video-assisted thoracic surgery, B transdiaphragmatic resection, C subxiphoid approach, OS overall survival, PFS progress-free survival, NA, not available

**Figure 3:**
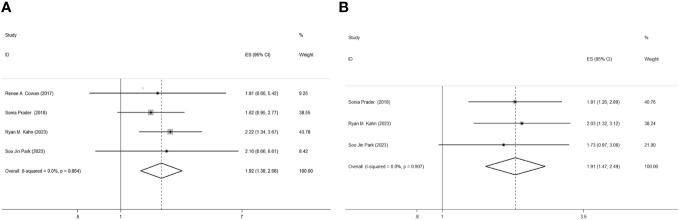
Forest plots of the association between residual disease and overall survival **(A)** and progress-free survival **(B)** with hazard ratio (HR) and 95% confidence interval (CI).

The experimental group included patients with residual disease in the abdominopelvic or thoracic region after PDS, while the control group included patients without residual disease in both the abdominopelvic and thoracic regions. All the studies provided information on OS. The heterogeneity was low (I^2^ = 0.0%, p=0.864), and we used a fixed-effects model to evaluate the combined OS, which suggested that residual disease predicted a worse OS with a combined hazard ratio (HR) of 1.92 (95% CI 1.38-2.68) (**Figure 3A**). Only three studies reported PFS information, and we used a fixed-effects model with no heterogeneity (I^2^ = 0.0%, p=0.907), which revealed that residual disease was also a risk factor for shortened PFS (HR=1.91, 95% CI=1.47-2.49) (**Figure 3B**).

### 3.3 Sensitivity analysis and publication bias

**Figure 4:**
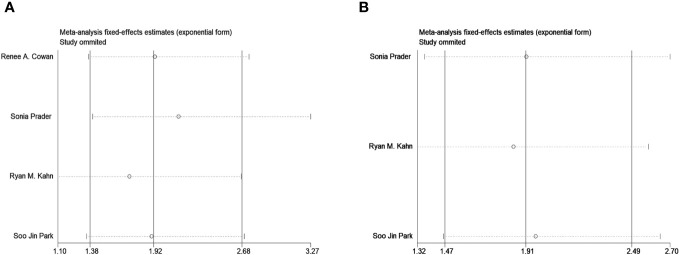
Sensitivity analysis plot on overall survival **(A)** and progress-free survival **(B)**.

**Figure 5:**
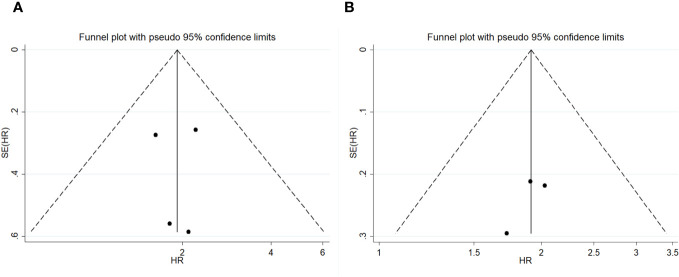
Funnel plot on overall survival **(A)** and progress-free survival **(B)**.

We also conducted sensitivity analysis for OS and PFS and found that omitting one study at a time did not lead to apparent fluctuations (**Figure 4**). A funnel plot is shown in **Figure 5**, and Egger’s and Begg’s tests for OS (Egger’s test p=1.00; Begg’s test p=0.982) and PFS (Egger’s test p=1.00; Begg’s test p=0.359) suggested no potential publications in the meta-analysis.

## 4 Discussion

We included 4 retrospective cohort studies in our meta-analysis, with a combined hazard ratio (HR)=1.92 (95% CI 1.38-2.68) for OS and a combined hazard ratio (HR)=1.91 (95% CI 1.47-2.49) for PFS, suggesting that maximal effort at cytoreduction may improve survival outcomes for advanced ovarian cancer patients. Only four studies were included in our analysis because of low heterogeneity; therefore, we did not perform subgroup analysis or meta-regression analysis to determine the origin of heterogeneity. However, the definition of residual disease was different in four articles. Park et al. (14) compared the sizes of residual tumors in abdominal and supradiaphragmatic areas ≥5 mm and <5 mm after PDS and investigated residual disease as a factor affecting poor PFS and OS. Similarly, Prader et al. (12) compared patients with a diameter of thoracic residual disease ≥5 mm and <5 mm after abdominal cytoreduction surgery who achieved no gross residual disease. In addition, Cowan et al. (11) and Kahn et al. (13) compared patients with no gross residual disease and a residual disease diameter of 1 cm. We performed a subgroup analysis to assess the impact of different cutoff values on survival outcomes (**Supplementary File 2**), and the hazard ratio (HR) for studies comparing patients with a diameter of 1 cm was 2.14 (95% CI 1.36-3.39), while the HR for another subgroup was 1.70 (95% CI 1.04-2.76), suggesting that residual disease is associated with worse OS. Similarly, PFS data from the other subgroups also revealed that residual disease was related to worse PFS. A number of studies have suggested that achieving no residual disease during upper abdominal complex debulking surgery improves survival outcomes with acceptable complications, especially during diaphragmatic and hepatobiliary disease resection (15, 16). Considering the risk and safety of debulking surgery, it is crucial to evaluate and select appropriate patients. In addition, Vizzielli et al. calculated a simple adjusted laparoscopic score to predict major postoperative complications after PDS, which could help surgeons adopt tailored strategies on an individual basis (17). In addition, a number of randomized trials which compared survival outcomes of neoadjuvant chemotherapy (NACT) and PDS suggested that survival outcomes in neoadjuvant chemotherapy followed by interval debulking surgery (IDS) was not inferior to PDS followed by chemotherapy with no significant difference in global quality-of-life (QoL) (18–21). Considering these findings, NACT or PDS should be carefully selected, and maximal effort at performing cytoreduction both in the intrathoracic and abdominopelvic tract may improve survival outcomes.

To our knowledge, the role of lymphadenectomy in advanced ovarian cancer is controversial. Panici et al. reviewed recent evidence and reported that no better outcomes or higher complication and mortality rates were associated with lymphadenectomy according to clinical trials (22). With the extension of treatment to thoracic cytoreductive surgery, a growing number of studies have reported the relationship between the CPLN and supradiaphragmatic lymph node (SPLN) and the prognosis in advanced ovarian cancer patients. Although the detailed mechanism of lymphatic drainage is largely unknown, it has been proposed that the main routes run through the right diaphragm to the CPLN or SPLN (23, 24). An increasing number of studies have evaluated the relationship between an enlarged CPLN and patient prognosis. Kolev et al. (25) performed a retrospective study that included 212 epithelial ovarian cancer patients with FIGO III to IV disease who had preoperative computed tomography (CT) scans and who underwent PDS. For the 155 patients who underwent optimal cytoreduction, the median survival was 5 months shorter than that of patients with enlarged SPLNs (p=0.09), suggesting a trend toward worse survival in patients with enlarged SPLNs. Similarly, Song et al. (26) found a significant negative influence on PFS in patients with enlarged SPLNs and no residual disease after PDS. In addition, McIntosh et al. (27) Raban et al. (28) and Luger et al. (29) also demonstrated a relationship between the SPLN and worse PFS and OS. However, Plana et al. (30) conducted a retrospective study that included 208 advanced ovarian cancer patients who underwent PDS and did not find any association with survival or an enlarged CPLN on CT. The strongest prognostic factor for OS in advanced ovarian cancer patients is residual disease. However, the relationship between enlarged CPLNs/SPLNs on CT and patient prognosis remains controversial, and previous studies have shown that complete cytoreduction surgery via the abdomen may improve survival outcomes in patients with advanced ovarian cancer but not in patients with residual thoracic disease (29, 31). As a result, when thoracic lesions are found in advanced ovarian cancer patients, whether surgeons perform intrathoracic cytoreduction and whether surgery is safe remain controversial. Boerner et al. (32) conducted a retrospective cohort study that included 100 advanced ovarian cancer patients with moderate-to-large pleural effusions who underwent VATS, and the results suggested that macroscopic intrathoracic disease was independently associated with increased risk of death (HR 2.18, 95% CI 1.14-4.18; p = .019). Patients who achieved no residual disease at the time of VATS or PDS had the best outcome. However, Lee et al. (33) identified 295 advanced epithelial ovarian cancer patients who underwent ^18^F-FDG positron emission tomography/computed tomography (PET/CT). Compared to patients with PET/CT stage III disease, patients with PET/CT IV disease and an enlarged SPLN had significantly worse OS (p=0.016) and PFS (p < 0.001). However, resection of SPLN lesions did not improve survival outcomes. In addition, Lee did not perform a subgroup analysis to evaluate the resection of the SPLN or survival according to residual disease. To our knowledge, this is the first meta-analysis on the relationship between residual disease resulting from intrathoracic or abdominopelvic cytoreductive surgery and patient prognosis, suggesting that advanced ovarian cancer patients may benefit from maximal cytoreduction in the intrathoracic or abdominopelvic region.

Intrathoracic cytoreduction surgery is usually performed by thoracic surgeons via VATS or gynecologic surgeons via a transdiaphragmatic approach. Among the 490 patients included in the meta-analysis, only 19 experienced major postoperative complications, as reported by Cowan (11); 4 of these complications were pulmonary and considered intrathoracic surgery-related. No postoperative deaths were reported for 490 patients. In addition, early attempts at intrathoracic surgery have been reported in the literature. Kuusela et al. (34) compared postoperative complications during the period of ultraradical surgery (2016-2019), which included intrathoracic surgeries, to surgeries in 2013-2016, and the results suggested greater estimated blood loss (P<0.001), duration of surgery (P<0.001) and complications (P=0.001). However, compared to those in the ultraradical and conventional radical surgery subgroups, the rate of major complications was not significantly different (p =0.234). Lopes et al. (35) retrospectively reviewed advanced epithelial ovarian cancer patients who underwent CPLN resection during cytoreduction surgery via the transdiaphragmatic approach and found acceptable complications. The most common pulmonary atelectasis was noncomplicated; however, no postoperative deaths or other CPLN-related complications were reported. Similarly, Yoo et al. (36) reported no specific intraoperative complications in 11 advanced ovarian cancer patients who underwent CPLN resection via the transabdominal approach. Recently, Nasser et al. (37) performed a systematic review of intrathoracic cytoreduction surgery via VATS or transdiaphragmatic outcomes in FIGO IV epithelial ovarian cancer patients; 9 studies ranging from 2007 to 2017 were included, and no thoracic cytoreduction surgery-related deaths were investigated. Only one patient experienced pneumothorax. In conclusion, with the attempt at intrathoracic surgery by thoracic surgeons and gynecologic surgeons, both of these approaches seemed to be feasible and associated with acceptable morbidity.

To our knowledge, this is the first meta-analysis evaluating the benefit of intrathoracic and abdominal cytoreduction surgery in ovarian cancer patients. Nonetheless, our systematic review and meta-analysis had certain limitations. First, the main limitation was the quantity and quality of the included studies. We included only 4 studies with different cutoff values, and all of them were retrospective cohort studies, which may lack representativeness and cause publication bias. In addition, only studies in English were included, and studies in other languages were missing, which increased publication bias. In addition, the statistical analysis used to evaluate publication bias involved Egger’s and Begg’s tests, which have lower statistical power when the number of studies is less than 10.

## 5 Conclusions

In conclusion, our systematic review and meta-analysis indicated that maximal cytoreduction in the intrathoracic and abdominopelvic tract may improve survival outcomes in advanced ovarian cancer patients with acceptable complications. Additionally, further large-scale, multicenter, randomized controlled studies are needed.

## Data availability statement

The original contributions presented in the study are included in the article/**Supplementary Material**. Further inquiries can be directed to the corresponding author.

## Author contributions

JW: Data curation, Methodology, Writing – original draft, Writing – review & editing. XW: Data curation, Writing – review & editing. WY: Methodology, Writing – review & editing. SZ: Data curation, Writing – review & editing.

## Supplementary material

Supplementary File 1 The search strategy for PubMed

Supplementary File 2 Forest plots of the subgroup analysis of cut-off value and overall survival **(A)** and progress-free survival **(B)** with hazard ratio (HR) and 95% confidence interval (CI).
