# Supplementary material for: Survival impact and safety of intrathoracic and abdominopelvic cytoreductive surgery in advanced ovarian cancer: a systematic review and meta-analysis
Source: Front Oncol. 2024 Jan 18;14:1335883. doi: 10.3389/fonc.2024.1335883 (PMC10830636; doi:10.3389/fonc.2024.1335883)
Supplement: Supplementary File 1 — The search strategy for PubMed [file DataSheet_1.pdf]

Supplemental File 1 The search strategy for PubMed.

Query	Search term
#1	((((((((((((((Ovarian Neoplasm) OR (Ovary Neoplasm)) OR (Ovary Cancer)) OR (Ovarian Cancer)) OR (Cancer of Ovary)) OR (carcinogenesis of the ovary)) OR (malignancy of the ovary)) OR (malignant neoplasm of the ovary)) OR (malignant ovarian neoplasm)) OR (malignant ovarian tumor)) OR (malignant ovary tumor)) OR (malignant tumor of the ovary)) OR (ovarial cancer)) OR (ovarian carcinogenesis)) OR (ovarian malignancy)) OR (ovary carcinogenesis)
#2	(((((((Cytoreduction Surgical Procedure) OR (Debulking Surgical Procedure)) OR (Cytoreductive Surgery)) OR (Cytoreductive Surgical Procedure)) OR (Debulking surgery)) OR (Debulking procedure)) OR (Cytoreductive procedure)) OR (ultraradical)
#3	((((((((((((((Thorax) OR (Thorace)) OR (Chest)) OR (Mediastinum)) OR (Pleura Visceral)) OR (Pleura Parietal)) OR (Pleura)) OR (Pleurorrhea)) OR (pleural effusion)) OR (pleura effusion)) OR (Cardiophrenic lymph node)) OR (Precordial lymph node)) OR (Paracardial lymph node)) OR (Supradiaphragmatic)) OR (mediastinal lymph node)) OR (mediastinum lymph node) OR (video assisted thoracoscopic surgery)) OR (thoracoscopy)
#4	#1 AND #2 AND #3
