# Supplementary material for: Survival impact and safety of intrathoracic and abdominopelvic cytoreductive surgery in advanced ovarian cancer: a systematic review and meta-analysis
Source: Front Oncol. 2024 Jan 18;14:1335883. doi: 10.3389/fonc.2024.1335883 (PMC10830636; doi:10.3389/fonc.2024.1335883)
Supplement: Supplementary File 2 — Forest plots of the subgroup analysis of cut-off value and overall survival (A) and progress-free survival (B) with hazard ratio (HR) and 95% confidence interval (CI). [file DataSheet_2.pdf]

Supplemental File 2 Forest plots of the subgroup analysis of cut-off value and overall survival (A) and progress-free survival (B) with hazard ratio (HR) and 95% confidence interval (CI).

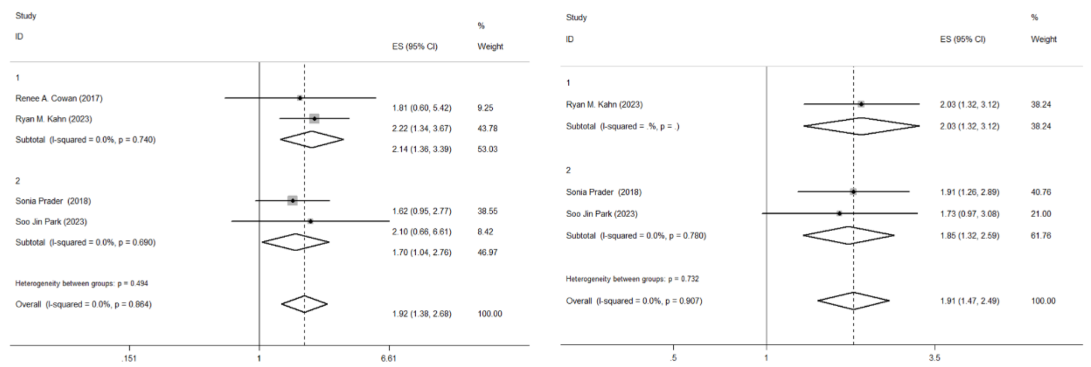

A B
